# N^6^-methyladenosine Reader IGF2BP2-modified HMMR Promotes Non-small Cell Lung Cancer Metastasis via Interaction with MAP4K4

**DOI:** 10.7150/ijbs.104097

**Published:** 2025-01-21

**Authors:** Jiansheng Zhang, Mengzhu Zhang, Aimin Qiu, Chang Li, Qiongju Chen, Jianjun Li, Yuanyuan Zeng, Jianjie Zhu, Jian-an Huang, Xiuqin Zhang, Zeyi Liu

**Affiliations:** 1 Department of Pulmonary and Critical Care Medicine, The First Affiliated Hospital of Soochow University, Suzhou, 215006, China.; 2 The Yancheng School of Clinical Medicine of Nanjing Medical University, Yancheng Third People's Hospital, The Affiliated Hospital of Jiangsu Vocational College of Medicine, Yancheng, 224000, China.; 3 Institute of Respiratory Diseases, Soochow University, Suzhou, 215006, China.; 4 Suzhou Key Laboratory for Respiratory Diseases, Suzhou, 215006, China.

**Keywords:** HMMR, MAP4K4, IGF2BP2, N^6^-methyladenosine, non-small cell lung cancer

## Abstract

Globally, lung cancer represents the leading cause of cancer-related mortality, with 85% of cases attributable to non-small cell lung cancer (NSCLC). Metastatic progression remains a major challenge in treating advanced lung cancer, resulting in a dismal five-year survival rate of 20-30%. Hyaluronan mediated motility receptor (HMMR) has been identified as a novel oncogene in NSCLC. However, its exact role and mechanisms in NSCLC and metastasis are yet to be fully understood. Elevated mRNA and protein levels of HMMR were observed in human NSCLC tumors in comparison with normal adjacent tissues. Increased HMMR expression was associated with poorer prognosis, with multivariate Cox regression analysis also identifying it as an independent prognostic factor. HMMR knockdown inhibited tumor cell migration and invasion, while its overexpression enhanced these processes. Mechanistically, HMMR promotes tumor metastasis by binding to mitogen-activated protein kinase kinase kinase kinase 4 (MAP4K4), which activates the p-JNK/p-c-JUN/MMP1 signaling cascade. The effects of HMMR overexpression on metastatic potential and JNK signaling were confirmed by MAP4K4 knockdown or GNE-495 treatment. Additionally, insulin like growth factor 2 mRNA binding protein 2 (IGF2BP2) was found to bind to the N^6^-methyladenosine (m^6^A) site of HMMR, increasing mRNA stability and HMMR expression levels. In a mouse model, the MAP4K4 inhibitor GNE-495 successfully suppressed lung metastasis induced by HMMR overexpression. These results offer valuable insights into HMMR's biological functions while suggesting potential avenues for novel treatments.

## Introduction

Lung cancer is one of the main causes of cancer-related deaths around the world, and as such, it represents a major challenge for public health^1^,^2^. Lung cancer develops through a multifaceted process involving various risk factors, including tobacco use, exposure to environmental toxins and genetic predispositions, all of which contribute to the disease's insidious progression^2^. NSCLC accounts for greater than 85% of all cases and it is estimated that 40% of NSCLC cases are adenocarcinoma, followed by 25% by squamous cell carcinoma^2^,^3^. Diagnoses are often made at advanced stages, complicating effective treatment. Although a range of therapeutic options are available, such as chemotherapy, radiotherapy, targeted therapies and immunotherapy^4^,^5^, managing metastasis in advanced lung cancer remains a significant challenge^6^, leading to poor prognoses and low survival rates^7^,^8^. Consequently, understanding the molecular mechanisms behind cancer cell metastasis has become a vital area of research in the development of effective lung cancer treatments.

Hyaluronan Mediated Motility Receptor (HMMR), also referred to as RHAMM or CD168, is crucial in processes such as cell growth, differentiation, and migration, mediated by hyaluronic acid^9^,^10^. It is characterized by a prominent coiled-coil (CC) structure that provides multiple sites for protein interactions^10^. HMMR functions as a spindle assembly factor, regulating dynein motor activity and thereby influencing cell division and the cell cycle^10^,^11^. Elevated HMMR expression has been identified as a biomarker in various tumors, with its presence linked to poor prognosis in lung^12^, hepatocellular^13^,^14^, breast^15^,^16^, colorectal^17^, pancreatic^18^ and prostate cancers^19^,^20^. Although the oncogenic role of HMMR is yet fully understood, its exact mechanism and function in lung cancer are still unclear, highlighting the need for further research to gain deeper insights into this area.

Genetic and epigenetic alterations enable cancer cells to acquire metastatic capabilities^21^. Among eukaryotic mRNAs, N^6^-methyladenosine (m^6^A) represents a predominant and conserved internal modification^22^ which is not only reversible but also regulated dynamically by m^6^A-binding proteins, or "readers," that influence the functions of m^6^A-modified RNAs^23^. These readers bind to m^6^A modification sites, affecting processes such as alternative splicing, mRNA stability, and translation^24^. The primary RNA-binding protein families involved are IGF2BPs and the YTH domain family. The latter, which includes YTHDF1-3, regulates m^6^A-mediated translation and degradation, while IGF2BPs (IGF2BP1-3) enhances the stability and translation of mRNA^22^,^23^. Disruption in the regulation of m^6^A writers, erasers, and readers contributes to cancer development by influencing oncogene and/or tumor suppressor expression, thus promoting cancer proliferation and metastasis^25^. Nevertheless, there is still a lack of consensus regarding the question of whether HMMR is subjected to m^6^A modification and the precise regulatory mechanisms involved in this process in NSCLC.

This work aimed to clarify HMMR's role in the progression of NSCLC before uncovering the mechanisms underlying its effects. Our findings revealed that increased HMMR expression was linked to metastasis and poor prognosis in patients with lung adenocarcinoma (LUAD). Additionally, we discovered that IGF2BP2 contributes to the upregulation of HMMR through an m^6^A modification-dependent manner. Mechanistically, HMMR promotes metastasis by interacting with MAP4K4, which in turn activates the JNK signaling pathway. Furthermore, our results suggest that the MAP4K4 inhibitor GNE-495 could potentially inhibit metastasis *in vivo*, presenting a promising new approach for the treatment of NSCLC.

## Materials and Methods

### Culture of cell lines

NSCLC cell lines (H1299, H292, HCC827, and A549), sourced from Procell (Wuhan, China), were cultured in either RPMI 1640 medium or F12K medium, with both containing 1% penicillin-streptomycin (Invitrogen, USA) and 10% fetal bovine serum (Gibco, USA). The cultures were then maintained at 37°C in 5% CO2 humidified atmosphere. Each cell line was confirmed as being mycoplasma-free, and its authenticity was validated through quality assessments of morphology and growth characteristics.

### Tissue samples

Tissue microarrays (ZL-LugA961) were purchased from Weiao Biotechnology Co., Ltd (Shanghai, China) for the analysis of HMMR protein levels in 48 paired lung adenocarcinoma and adjacent normal tissues. The research was approved under ethics number ZLL-15-01 by the Shanghai Zhuoli Biotech Company.

### RNA interference

Various short interfering RNA (siRNA) sequences that target specific coding regions of the HMMR, MAP4K4, c-JUN, IGF2BP2, FTO and ALKBH5 genes were designed. These different siRNAs were then synthesized by GenePharma (Shanghai, China), with their target sequences provided in Table S1. This was followed by transient transfection of siRNAs into cells using Lipofectamine 2000 (Invitrogen, USA), with the successfully transfected cells eventually collected for subsequent analysis after 48-72 hours of incubation.

### RNA Extraction and Quantitative Reverse Transcription-Polymerase Chain Reaction (qRT-PCR)

Total RNA extraction was performed with TRIzol Reagent (Thermo Fisher Scientific, USA), followed by reverse transcription with Takara's MMLV Reverse Transcriptase to generate cDNA. PCR analysis was then conducted on an ABI Step One Plus Real-Time PCR system (Applied Biosystems Inc., USA) using the primer sequences listed in Table S2 as well as the TaKaRa SYBR® Premix Ex TaqTM kit (Osaka, Japan). β-actin was selected as the internal control, with the 2^-ΔΔCt^ method subsequently used to assess relative gene expression.

### Establishment of stable HMMR knockdown and overexpression cell lines

Lentiviruses for HMMR control, overexpression, and knockdown were obtained from GenePharma Corporation (Shanghai, China). To establish stable cell lines, the cells were subjected to 2 µg/mL puromycin (Sigma-Aldrich, USA), with the most resistant ones eventually isolated.

### Western blot analysis

Western blot was conducted in accordance with established procedures. Primary antibodies used were: anti-HMMR (15820-1-AP), anti-MAP4K4 (55247-1-AP), anti-IGF2BP2 (11601-1-AP), anti-ALKBH5 (16837-1-AP), anti-β-actin (81115-1-RR) from ProteinTech (Wuhan, China); anti-p-JNK (#4668), anti-c-JUN (#9165), anti-p-c-JUN (#3270) from CST (Cell Signaling Technology, USA); anti-JNK (#AF6318), anti-p-JNK (#AF3318), anti-MMP1 (#AF0209) from Affinity (Changzhou, China). Secondary antibodies used were anti-rabbit (CW0103S) and anti-mouse (CW0102S) from CWBIO (China).

### Wound healing assay

A549 and H1299 stable cells were allowed to reach about 70-80% confluence and form a monolayer in 6-well plates. A scratch was then made with a fresh10-µl pipette tip across the center of each well, thus forming a gap the same width as the tip. A perpendicular scratch was then made to create a cross-shaped pattern, and this was followed by a washing step, repeated twice with PBS, to remove detached cells. Fresh medium was then added to the wells, and after a 24-hour incubation, a microscope (CKX41, Olympus) was used with consistent magnification and settings to observe and record cell migration.

### Cell migration and invasion assays

For these assays, 24-well plates with Transwell inserts (8-µm pore size; Corning, USA) were used. For the migration assay, stable cells were added to FBS-free medium before a 24-hour incubation at 37 °C in the top chamber of Transwell insert. In addition, a volume of 800 µL of complete medium containing 10% serum should be added to the lower chamber. Cells that had migrated towards the inferior aspect of the insert were subjected to a 30-minute fixation period in 100% methanol. Following this, the cells were left to air-dry for 10 minutes prior to a 30-minute staining using 0.1% crystal violet. Regarding the invasion assay, Matrigel (BD Science, USA) diluted in serum-free medium was used to precoat the inserts before incubation for 2 hours at 37 °C. The remaining steps followed the migration assay protocol. A microscope (CKX41, Olympus) was eventually used to capture cell images, with at least three fields (magnification, ×100) counted. Each assay was performed in triplicate.

### mRNA sequencing

TRIzol Reagent (1 ml per 10 cm dish) was used to lyse H1299 cells, either vector-transfected or HMMR-overexpressing stable cells. RNA sequencing was conducted using an Illumina HiSeq XTen sequencer (Wuhan Maiteweier Biotechnology Co., Ltd., China). The analysis was conducted with a 3:3 ratio for the two groups.

### Mass spectrometry analysis

Cell lysates from H1299 Flag-HMMR-overexpressing stable cells and control cells were prepared using a mixture of a proteinase cocktail inhibitor (Roche, USA) and IP lysis buffer (Beyotime, China). Anti-Flag magnetic beads were then added to the lysates, which were sent to Wuhan Maiteweier Biotechnology Co. for label-free proteomics analysis.

### Co-immunoprecipitation (Co-IP)

A 10-cm plate of A549 or H1299 cells at 95-100% confluency was used to assess endogenous Co-IP. The cells were detached by scraping, after which 1 ml of modified IP lysis buffer (Beyotime, China), containing a cocktail of phosphatase and protease inhibitors (Sigma-Aldrich, USA), was used for 30 minutes of cell lysis at 4°C. The lysate was then subjected to a 15-minute centrifugation at 12,000 g and 4°C, with the resulting supernatants incubated overnight with either IgG or specific antibodies against the protein of interest. Following incubation, 20 µL of protein A/G magnetic beads was added to the lysates, with 4 hours of gentle mixing at 4°C. The beads were washed three times with IP lysis buffer and then boiled in 1× protein loading buffer for 10 minutes at 100°C.

### Immunofluorescence staining

After seeding cells into 24-well plates containing glass slides, the cells were allowed to reach 40-50% confluency with a 24-hour incubation period. PBS was then used to wash the cells after which 20 minutes of cell fixation, followed by 20 minutes of cell permeabilization, were performed using 4% paraformaldehyde and 0.5% Triton X-100, respectively. Blocking was performed using 5% BSA prior to overnight incubation at 4°C with antibodies against p-c-JUN or MAP4K4. Detection was carried out using fluorescently labeled secondary antibodies (diluted 1:500, Beyotime Biotechnology). Counterstaining was performed with DAPI (Life Technologies) for 3 minutes. The visualization of fluorescent signals was conducted with the aid of a Leica SP8 confocal microscope.

### RNA-Binding Protein Immunoprecipitation (RIP)

NSCLC cells were allowed to reach 90% confluence in 10-cm plates, after which they were harvested and lysed on ice for 10 minutes using polysome lysis buffer containing RNase and protease inhibitors. The lysates were mixed by pipetting, stored at -80 °C for 5 minutes and thawed on ice prior to a 10-minutes centrifugation at 16100 g and 4°C. One part of the resulting supernatant was used for whole cell extraction (input group) while the other part was used for immunoprecipitation (IP group) which involved incubating the lysates overnight with either 5 μg anti-IGF2BP2 or IgG antibodies at 4 °C. Polysome lysis buffer was used to wash protein A/G magnetic twice before mixing them with the lysate-antibody complexes with 1-hour of continuous stirring at 4 °C. The RNA-protein complexes were then washed three times with elution buffer and treated with proteinase K at 55 °C for 1 hour. Extracted RNAs were analyzed by qRT-PCR, with relative enrichment eventually normalized to the input.

### Methylated RNA Immunoprecipitation (MeRIP)

After extracting total RNA as described before, 10% of the extracted sample was kept aside as the input control, while the remaining RNA was used for m^6^A-IP. The riboMeRIP™ m^6^A Transcriptome Profiling Kit was then used as required by the manufacturer to bind anti-m^6^A antibody or IgG to magnetic beads. The total RNA was then incubated with the antibody-conjugated beads in 500 μl binding buffer, with 2-hours of continuous stirring at 4 °C. m^6^A-modified mRNAs were eluted from the beads using elution buffer, and after purification, they were analyzed by qRT-PCR. Relative enrichment was normalized to the input. The primers for enrichment of m^6^A-modified mRNAs are provided as Table S3.

### RNA stability assays

NSCLC cells, plated in six-well plates, were incubated for 24 hours until an 80% confluency was reached. The cells were then treated with actinomycin D (10 µg/ml, Selleck, USA) before being harvested at 0, 4, 8 and 12 hours. This was followed by total RNA extraction, after which qRT-PCR was performed to estimate the degradation rate of mRNA according to established protocols.

### Luciferase reporter assays

After seeding NSCLC cells in 24-well plates, a 24-hour incubation was performed before transfection with plasmids containing wild-type or mutant HMMR-CDS. Forty-eight hours following transfection, the Dual Luciferase® Reporter Assay System (Vazyme Biotech Co., Ltd.) was employed to quantify Firefly and Renilla luciferase intensity.

### *In vivo* metastasis assays

The Laboratory Animal Centre of Soochow University provided 5-weeks old female BALB/c nude mice which were then housed in a pathogen-free environment. A LUAD metastasis model was established by injecting the mice intravenously with either HMMR overexpressing or control A549 cells (2 million cells/100 ul/mouse). From the fifth week post-injection, mice received daily intraperitoneal injections of either DMSO (3 mg/kg) or the MAP4K4 inhibitor GNE-495 (HY-100343, MCE, China) (3 mg/kg). Eight weeks after inoculation, the animals were euthanized, and their lungs were collected and fixed in Bouin's solution for macroscopic examination of metastatic nodules. Micrometastasis were evaluated by staining the lung tissues with hematoxylin and eosin (H&E). All experiments were performed according to the ethical guidelines of the Animal Ethics Committee of Soochow University (Ethics number 202402A0195).

### Bioinformatics analysis

RNAseq data from the TCGA database (https://portal.gdc.cancer.gov) were retrieved and combined with relevant clinical data from the LUAD project. The data were extracted in TPM format using STAR. Gene expression was then analyzed and visualized using the 'ggplot2' package of R software (version 4.2.1). Kaplan-Meier survival analysis was also conducted, and results were visualized with the 'survminer' and 'survival' packages. Multivariate Cox regression analysis was employed to identify variables for constructing a nomogram that predicts one-, three-, and five-year overall survival rates based on Cox proportional hazards models. The nomogram calculates recurrence risk by aggregating points for each risk factor using R's 'rms' package. Additionally, m^6^A modification sites were predicted for RNA sequences of interest using the SRAMP tool (http://www.cuilab.cn/sramp/).

### Statistical analysis

GraphPad Prism (version 8.0, GraphPad Software, USA) was used to statistically analyze all data which were presented as the mean ± SD of at least three independent replicates. The Chi-Square test examined the relationship between HMMR expression and clinicopathologic parameters. Kaplan-Meier survival curves were created, and Cox regression analysis assessed differences in survival probabilities between groups. Two-group and multiple-group comparisons were then analyzed using two-tailed Student's t-tests and one-way/two-way ANOVA, respectively. Results were considered to be statistically significant at *P* < 0.05, with *P < 0.05, **P < 0.01, ***P < 0.001, with ns indicating the absence of statistical significance.

## Results

### Expression and clinical significance of HMMR in NSCLC

**Figure 1:**
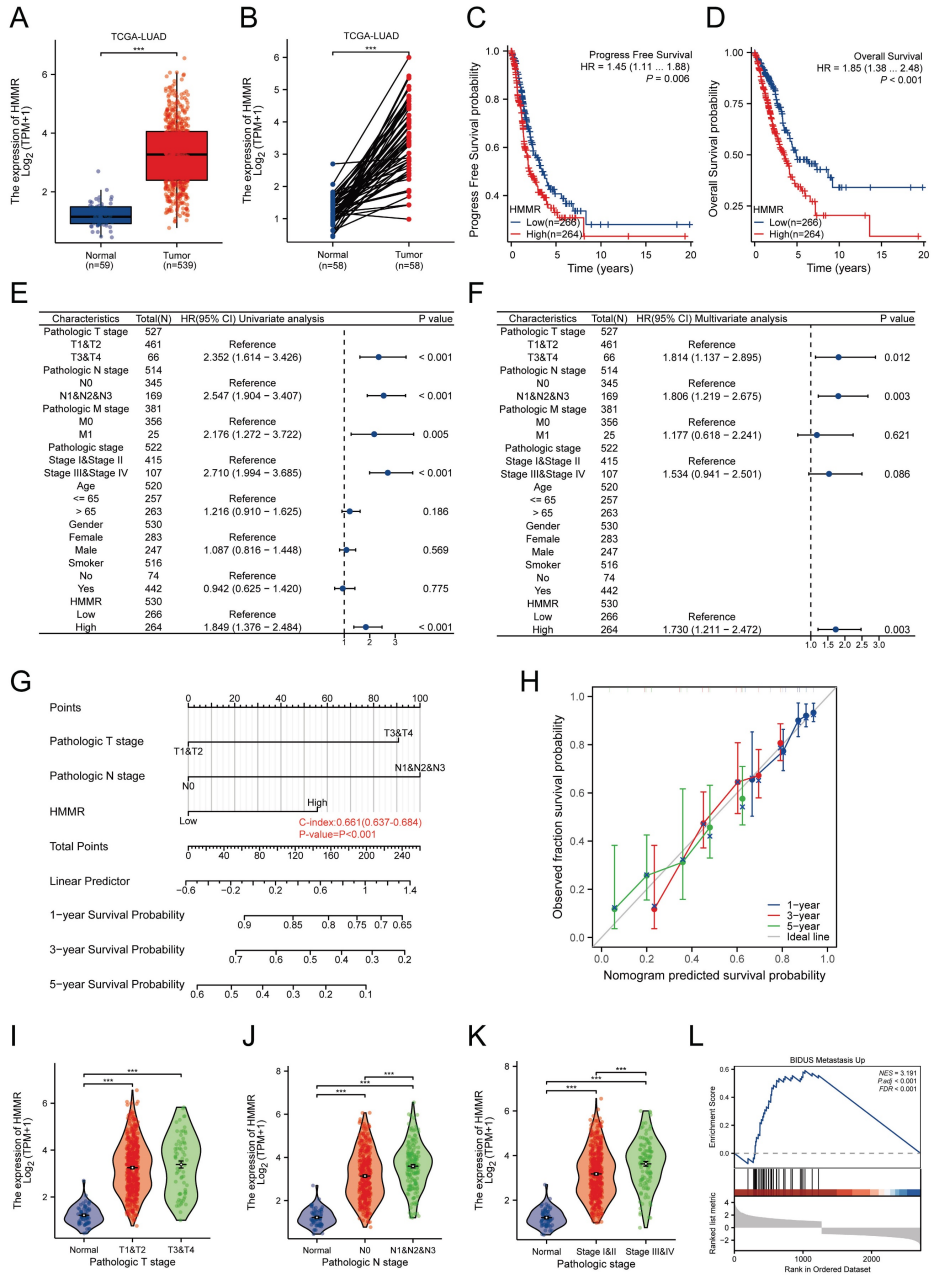
** Expression and clinical significance of HMMR in NSCLC.** (A and B) The TCGA database was utilized to compare the mRNA expression levels of HMMR in unpaired and paired LUAD tissues relative to normal lung tissues. (C and D) Analysis of progression-free survival (PFS) and overall survival (OS) probabilities in LUAD patients with high versus low HMMR expression, using the median expression level of HMMR as the cut-off value. (E and F) Univariate and multivariate Cox regression analyses were performed to identify independent prognostic factors involving HMMR and various clinical parameters. (G) A nomogram was developed to predict OS at 1, 3, and 5 years for LUAD patients. (H) The calibration curve for the OS nomogram model, where the ideal nomogram is represented by a dashed diagonal line, and the blue, red, and green lines indicate the observed nomograms for 1, 3, and 5 years, respectively. (I-K) Correlation analysis of HMMR expression levels with tumor (T) stage, nodal (N) stage, and pathological stage. (L) Gene Set Enrichment Analysis (GSEA) of altered signaling pathways in LUAD tissues, based on differentially expressed genes (DEGs) associated with high versus low HMMR expression.

To identify key genes associated with lung cancer, two GEO datasets (GSE21933 and GSE40275) were analyzed. Analysis revealed 794 differentially expressed genes (DEGs) in GSE21933 (297 upregulated and 497 downregulated) Figure S1A) and 588 DEGs in GSE40275 (265 upregulated and 323 downregulated) (Figure S1B). Both datasets also had 203 DEGs (74 upregulated and 129 downregulated) in common (Figure S1C). The PPI network for these overlapping DEGs was constructed using the STRING database (Figure S1D). Using the Cytoscape plugin MCODE, we identified the most significant clustering module with 57 nodes and 1463 edges (score: 52.25) (Figure S1E). The CytoHubba plugin, which identifies highly connected nodes, was then used to determine the top 10 hub genes: HMMR, PBK, CDK1, NUSAP1, KIF2C, MELK, TPX2, CCNA2, CCNB2, and CENPF (Figure S1F). Univariate Cox regression analysis indicated that HMMR is a risk factor for lung adenocarcinoma patients (Figure S1G). Elevated HMMR levels were observed in both unpaired and paired lung adenocarcinoma tissues in comparison with normal ones (Figure 1A-B). Kaplan-Meier survival analysis of TCGA data identified correlations between higher HMMR expression, reduced progression-free survival (PFS) and overall survival (OS) in LUAD patients, suggesting its potential as a prognostic marker (Figure 1C-D). Univariate Cox regression analysis identified pathological T stage, N stage, M stage, pathological stage, and HMMR as risk factors in LUAD patients (Figure 1E), while multivariate Cox regression analysis also found HMMR to be an independent prognostic factor (Figure 1F). Then we developed a nomogram incorporating HMMR, pathological T stage, and N stage for predicting the 1-, 3-, and 5-year survival probabilities for LUAD patients (Figure 1G). Points were assigned for each prognostic factor, with higher total points indicating poorer outcomes. The calibration curve demonstrated the nomogram's efficacy in estimating survival probabilities at 1, 3, and 5 years (Figure 1H). Furthermore, there was a strong association between the mRNA expression of HMMR and the N stage (Figure 1J) and pathological stage (Figure 1K), but no correlation was found with the T stage (Figure 1I). Gene Set Enrichment Analysis (GSEA) revealed that metastasis was the most significantly enriched pathway related to HMMR (Figure 1L). These results demonstrate a significant elevation in HMMR expression in lung cancer tissues, which correlates with an unfavourable patient prognosis and may serve as an independent prognostic indicator.

### High HMMR expression in LUAD tissues and its involvement in metastasis

**Figure 2:**
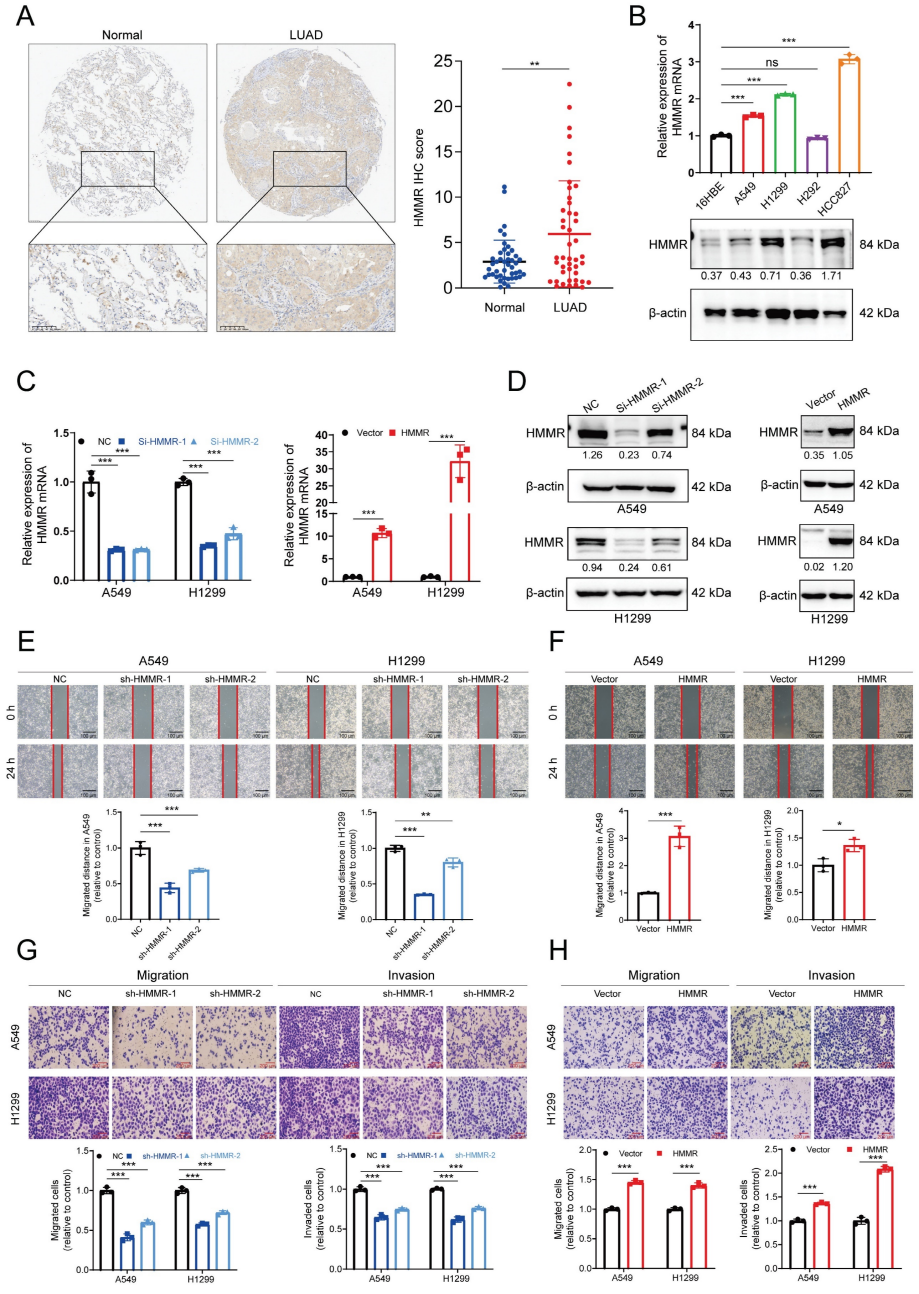
** High HMMR expression in LUAD tissues and its involvement in metastasis.** (A) Immunohistochemical images demonstrating HMMR expression in paired normal and LUAD tissues (n = 48). (B) qRT-PCR and Western blot analyses of HMMR expression in NSCLC cell lines and the normal bronchial epithelial cell, 16HBE. (C and D) Validation of HMMR knockdown and overexpression using qRT-PCR and Western blot at both mRNA and protein levels in A549 and H1299 cells, with β-actin as an internal control. (E-H) Wound healing and transwell assays depicting the mobility and invasiveness of NSCLC cells following HMMR knockdown or overexpression, with statistical data on migrated and invaded cell counts shown in the bottom panel. All data are presented as mean ± SD from three independent experiments.

Subsequently, immunohistochemistry (IHC) was employed to evaluate HMMR expression in 48 LUAD patient samples. The IHC analysis demonstrated higher HMMR expression in LUAD tissues in comparison with adjacent normal ones (Figure 2A). Subsequent correlation with clinicopathological characteristics highlighted significant associations between HMMR expression and the pathological N stage, M stage as well as the overall pathological stage (Table 1. Additionally, the mRNA and protein levels of HMMR were analyzed for 16HBE and four NSCLC cell lines. Compared with the normal cells, the NSCLC ones exhibited elevated HMMR levels, except in the case of H292 (Figure 2B). To investigate whether HMMR affects the migration and invasion capabilities of NSCLC cells, lentiviruses were used for transfecting A549 and H1299 cells with two different small interfering RNAs targeting HMMR or stably overexpressed HMMR. The successful silencing or overexpression of HMMR mRNA and protein was then confirmed by qRT-PCR and western blot (Figure 2C-D). Wound healing and Transwell assays further validated these findings, showing that HMMR silencing reduced cell mobility and invasiveness (Figure 2E, 2G), whereas HMMR overexpression enhanced the migration and invasion of cells (Figure 2F, 2H). Overall, the findings suggest that HMMR can promote the migration and invasion of NSCLC cells.

### HMMR regulates the JNK signaling pathway in NSCLC cell lines

**Figure 3:**
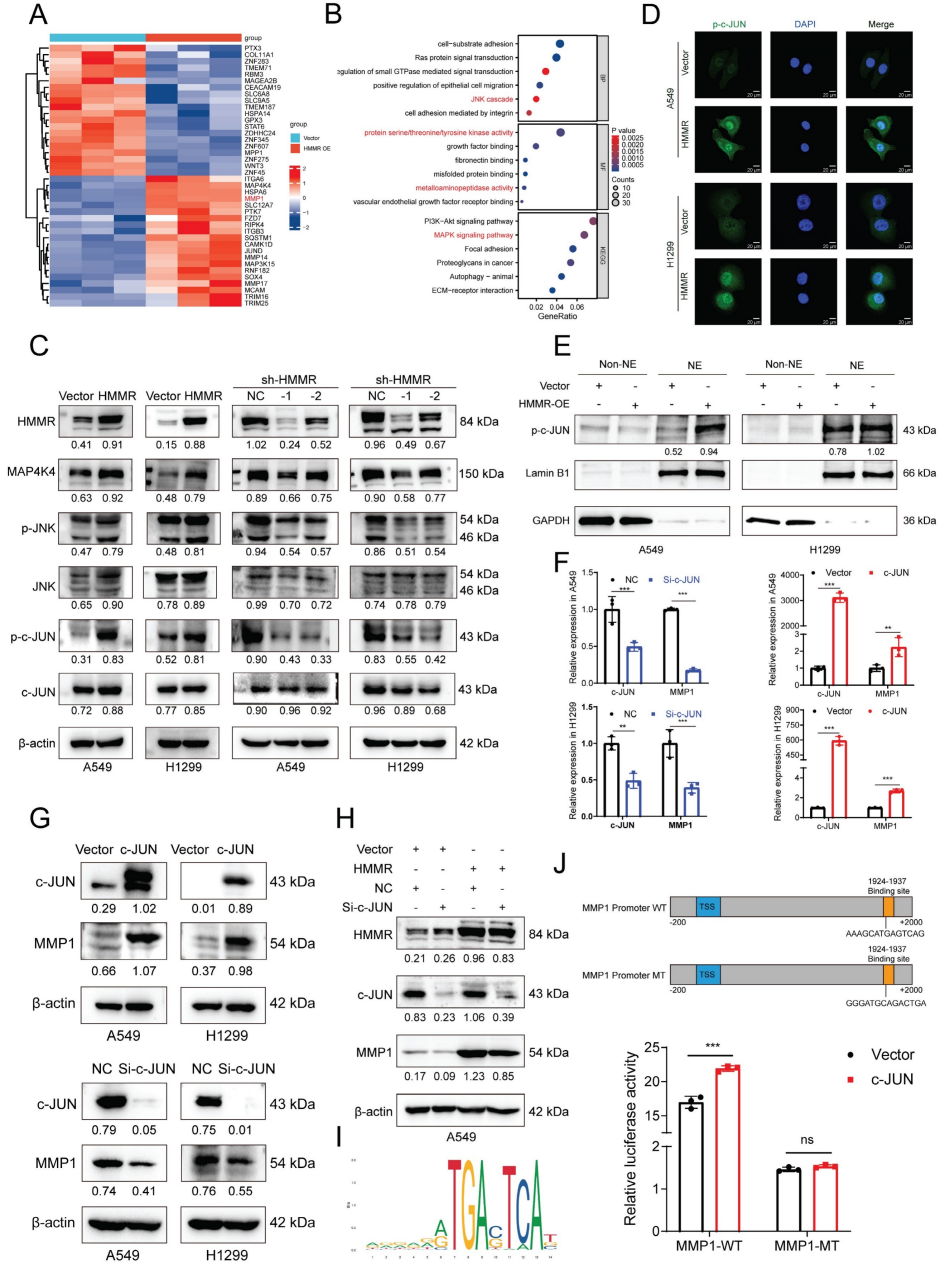
** HMMR regulates the JNK signaling pathway in NSCLC cell lines.** (A) Heatmap displaying mRNA expression levels in H1299 cells with vector control versus HMMR overexpression. (B) GO and KEGG enrichment analyses of HMMR-associated DEGs highlighting enriched biological processes (BP), molecular functions (MF), and KEGG pathways. (C) Western blot analysis showing protein levels of MAP4K4, p-JNK, JNK, p-c-JUN, and c-JUN in cells with HMMR overexpression or knockdown compared to control cells. (D) Immunofluorescence staining of p-c-JUN in HMMR overexpressing cells versus control cells. (E) Nucleocytoplasmic separation experiments showed more p-c-JUN nuclear accumulation in HMMR overexpressing cells compared to the vector cells. (F and G) qRT-PCR and Western blot analyses of MMP1 expression following transfection with either c-JUN plasmid or si-c-JUN. (H) Knockdown of c-JUN mitigates HMMR-induced upregulation of MMP1. (I) Prediction of the c-JUN binding region to the MMP1 promoter using the JASPAR website. (J) Relative luciferase activity of wild-type and mutant MMP1 reporter vectors in H1299 cells overexpressing c-JUN versus control cells.

To identify alterations in potential downstream signaling pathways, RNA sequencing was conducted on vector control and HMMR-overexpressing H1299 cells. The heatmap revealed that MMP1 levels were significantly increased in the HMMR overexpression group compared with the control (Figure 3A). Functional enrichment analyses of differentially expressed genes (DEGs) using GO and KEGG pathways showed that the biological process (BP) category was notably enriched in Ras protein signal transduction and the JNK cascade. The molecular function (MF) category was primarily enriched in protein serine/threonine/tyrosine kinase activity and metalloaminopeptidase activity. KEGG pathway analysis subsequently revealed that the MAPK signaling pathway, focal adhesion and ECM-receptor interaction pathways were significantly enriched (Figure 3B). The MAPK signaling pathway includes ERK, JNK, P38, and ERK5 pathways. Western blot analysis of p-JNK, JNK, and other key signaling molecules (p-c-JUN, c-JUN) demonstrated that HMMR overexpression significantly increased these signaling molecules, while HMMR knockdown resulted in a substantial decrease (Figure 3C). Immunofluorescence staining and nucleocytoplasmic separation experiments showed more p-c-JUN nuclear accumulation in HMMR overexpressed cells compared to the vector (Figure 3D-E). Furthermore, overexpression of c-JUN led to increased MMP1 expression, whereas c-JUN knockdown resulted in decreased MMP1 levels (Figure 3F-G). Subsequent rescue experiments revealed that interfering with c-JUN effectively counteracts the increase in MMP1 induced by HMMR overexpression (Figure 3H). Our findings indicate that p-c-JUN acts as a transcription factor, translocating to the nucleus in response to elevated HMMR levels (Figure 3D-E). To identify the binding site of c-JUN on the MMP1 promoter, we employed the JASPAR website for predictive analysis (Figure 3I). Luciferase assays demonstrated that c-JUN overexpression enhances MMP1 promoter activity, while mutations in the binding sites led to reduced luciferase activity (Figure 3J). These results support the hypothesis that HMMR facilitates NSCLC progression through the p-JNK/p-c-JUN/MMP1 signaling pathway. However, the exact mechanism through which HMMR activates JNK signaling remains elusive.

### HMMR-MAP4K4 interaction enhances NSCLC cell migration and invasion *in vitro*

**Figure 4:**
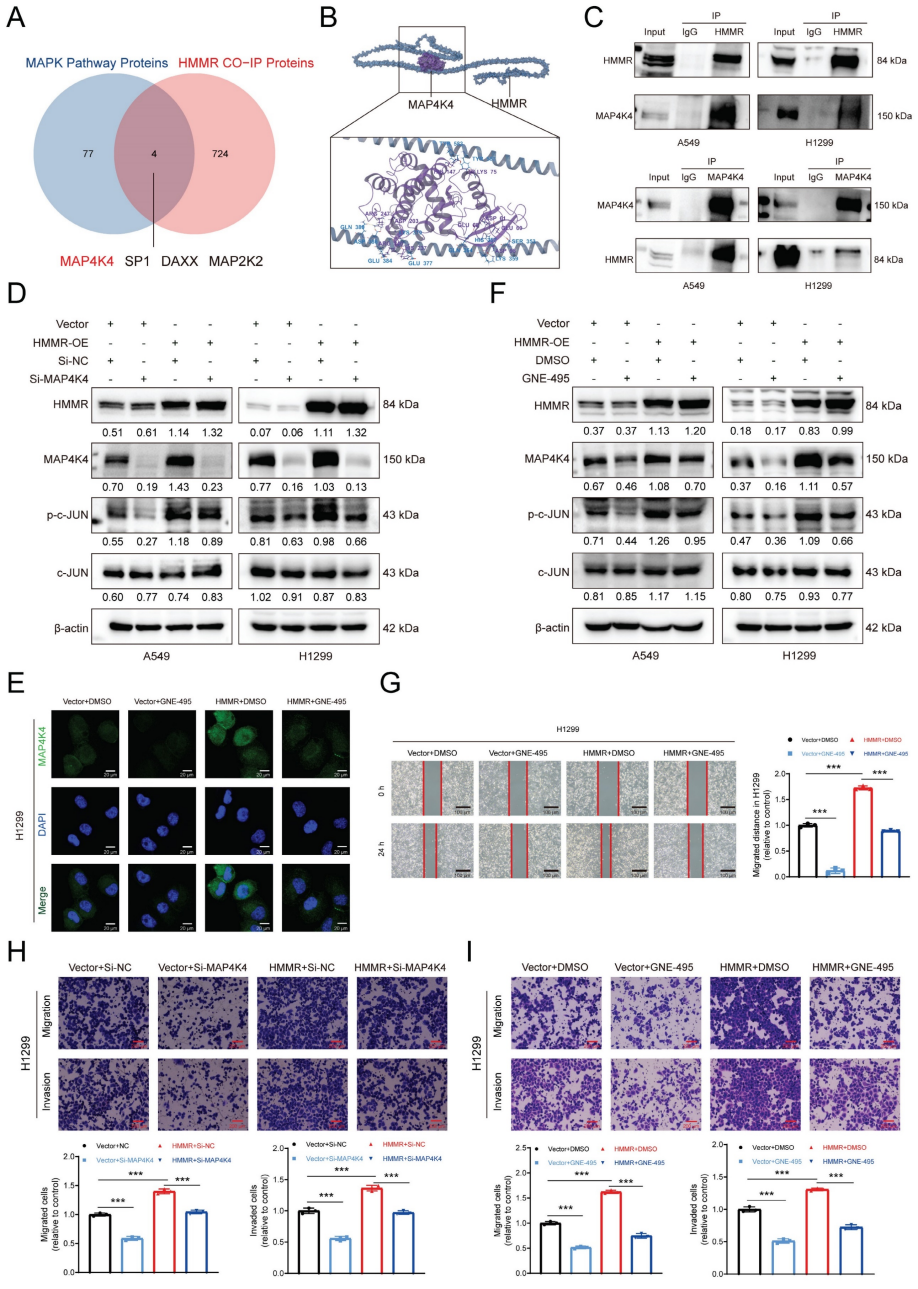
** HMMR-MAP4K4 interaction enhances NSCLC cell migration and invasion *in vitro*.** (A) Venn diagrams illustrating the overlap between HMMR-interacting proteins and those involved in the MAPK pathway. (B) Molecular modeling of the HMMR-MAP4K4 interaction. (C) Endogenous co-immunoprecipitation (co-IP) assays demonstrating interactions between HMMR and MAP4K4 in NSCLC cells. (D) Knockdown of MAP4K4 mitigates HMMR-induced upregulation of p-c-JUN in NSCLC cells. (E) Immunofluorescence staining of MAP4K4 in HMMR-overexpressing and control cells, with or without MAP4K4 inhibitor GNE-495. (F) The MAP4K4 inhibitor GNE-495 reduces HMMR-induced upregulation of MAP4K4 and p-c-JUN. (G-I) Knockdown of MAP4K4 or treatment with the MAP4K4 inhibitor GNE-495 attenuates HMMR-induced enhancement of migration and invasion.

The mechanism by which HMMR activates JNK signaling in NSCLC cells was elucidated by mass spectrometry analysis of Vector and HMMR-OE H1299 cells, with the results identifying 728 potential HMMR-interacting proteins. Since HMMR overexpression activates the JNK pathway, we cross-referenced 81 MAPK signaling-related proteins from the GSEA database with our list of candidate proteins, revealing four potential HMMR interactors (Figure 4A). KEGG database searches indicated that MAP4K4 is positioned upstream in the JNK signaling pathway. Molecular modeling further supported HMMR's interaction with MAP4K4 (Figure 4B). Additionally, TCGA-LUAD data found that MAP4K4 and HMMR expressions were positively correlated Figure S2A). Endogenous co-immunoprecipitation experiments further showed that HMMR interacted with MAP4K4 (Figure 4C).

Elevated MAP4K4 levels were observed in lung adenocarcinoma tissues compared with normal ones, with the MAP4K4 expression being also linked to poor patient prognosis as indicated by PFS and OS (Figure S2B-D). To investigate the HMMR/MAP4K4/p-c-JUN regulatory axis in NSCLC, rescue experiments were performed. Transfection of MAP4K4 siRNA into HMMR-overexpressing and vector cells restored abnormal MAP4K4 and p-c-JUN levels (Figure 4D). Additionally, transwell assays showed that reduced MAP4K4 expression mitigates the enhanced migratory and invasive capabilities resulting from HMMR overexpression (Figure 4H, S2G). Further exploration included testing MAP4K4 inhibitors as potential treatment strategies. GNE-495, a potent and selective MAP4K4 inhibitor, was applied to HMMR overexpressing and vector cells. Treatment with 5 µM GNE-495 for 48 hours significantly decreased MAP4K4 levels and c-JUN phosphorylation (Figure 4E-F, S2E). Wound healing and transwell assays demonstrated that the MAP4K4 inhibitor effectively reversed the increased migratory and invasive properties induced by HMMR overexpression (Figure 4G, 4I, S2F, S2H). Notably, interference with c-JUN also resulted in decreased mRNA and protein levels of MAP4K4 (Figure S2I, S2J). In summary, our data demonstrate that HMMR interacts with MAP4K4 to activate the p-JNK/p-c-JUN signaling pathway, thereby enhancing NSCLC cell metastasis. This effect can be reversed by the administration of either Si-MAP4K4 or the MAP4K4 inhibitor GNE-495.

### MAP4K4 inhibitor GNE-495 reduces NSCLC cell metastasis *in vivo*

**Figure 5:**
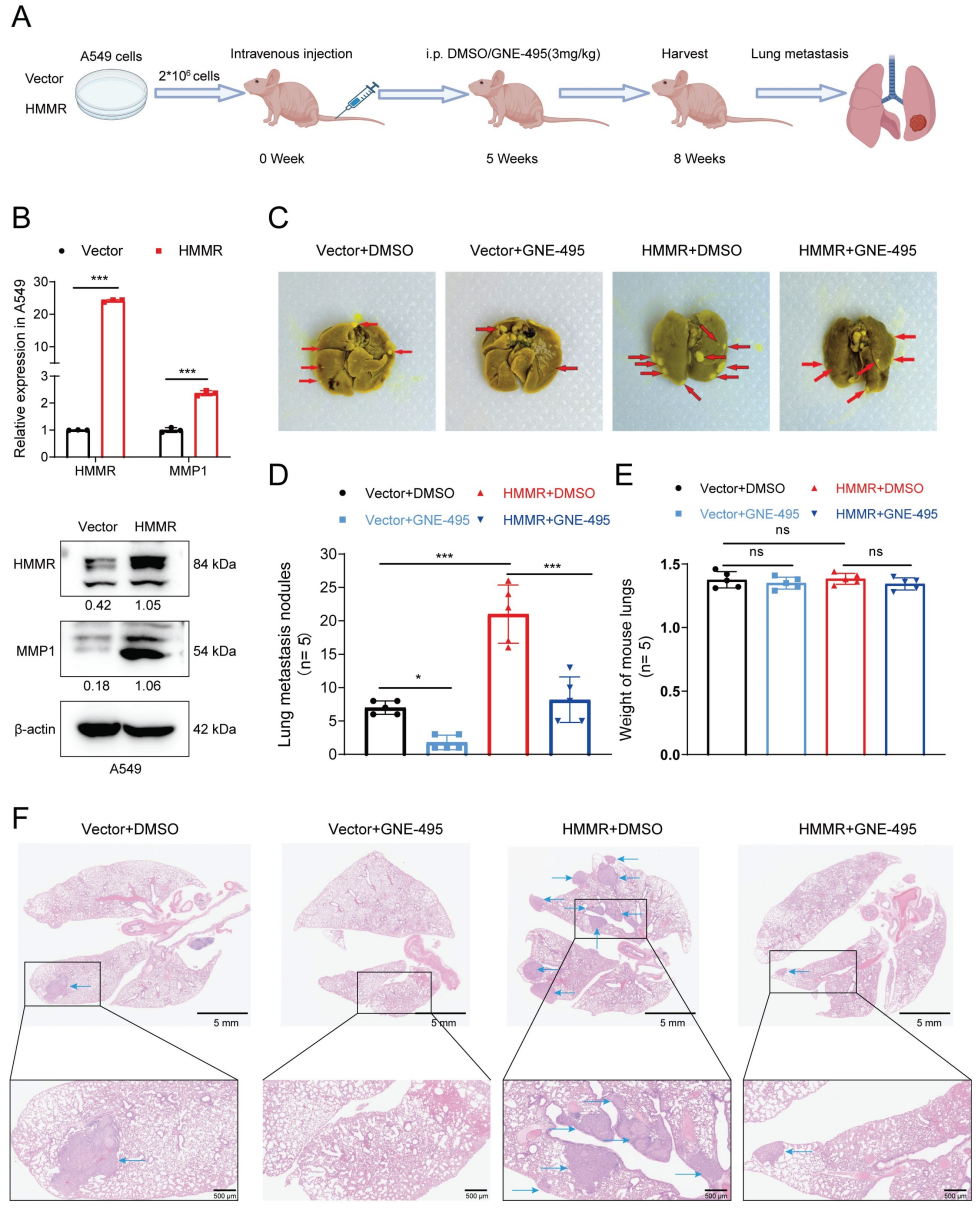
** MAP4K4 inhibitor GNE-495 reduces NSCLC cell metastasis *in vivo*.** (A) Flowchart illustrating the NSCLC cell *in vivo* metastasis model. (B) Relative mRNA and protein expression levels of HMMR and MMP1 in the A549 cells used to establish the *in vivo* metastasis model. (C) Photographs showing lung metastatic nodules in mice following injection of A549 cells overexpressing HMMR or the control vector, with treatment by DMSO or GNE-495. Metastatic nodules are indicated by red arrowheads. (D) Quantification of lung metastatic nodules across the four experimental groups. (E) The lung weights of the mice in each group after execution. (F) Hematoxylin and eosin (H&E) staining of lung tissue to assess micrometastatic foci. Representative histological images of micrometastatic foci in the four groups. Micrometastatic foci are indicated by blue arrowheads.

To assess HMMR's role in the metastasis of NSCLC cells, BALB/c nude mice were given intravenous injections of HMMR-overexpressing A549 cells or control cells. From the fifth week post-injection, mice received daily intraperitoneal injections of DMSO or GNE-495 (3 mg/kg). The animals were then euthanized after eight weeks, with their lungs subsequently examined to identify metastatic lesions (Figure 5A). Prior to inoculation, the A549 cells were also reanalyzed to determine the mRNA and protein levels of HMMR and MMP1 (Figure 5B). Histological analysis revealed more metastatic lung nodules in mice injected with HMMR-overexpressing A549 cells compared with those that were given control cells (Figure 5C-D, 5F, S2K). Nevertheless, there was no statistically significant difference in lung weights between the experimental groups of mice (Figure 5E). Importantly, GNE-495 treatment effectively reversed the metastasis associated with HMMR overexpression. The results of this study indicate that MAP4K4 inhibition with GNE-495 is a promising strategy for preventing NSCLC metastasis and could be a valuable therapeutic approach for patients with elevated HMMR levels.

### IGF2BP2 enhances HMMR expression by stabilizing HMMR mRNA

**Figure 6:**
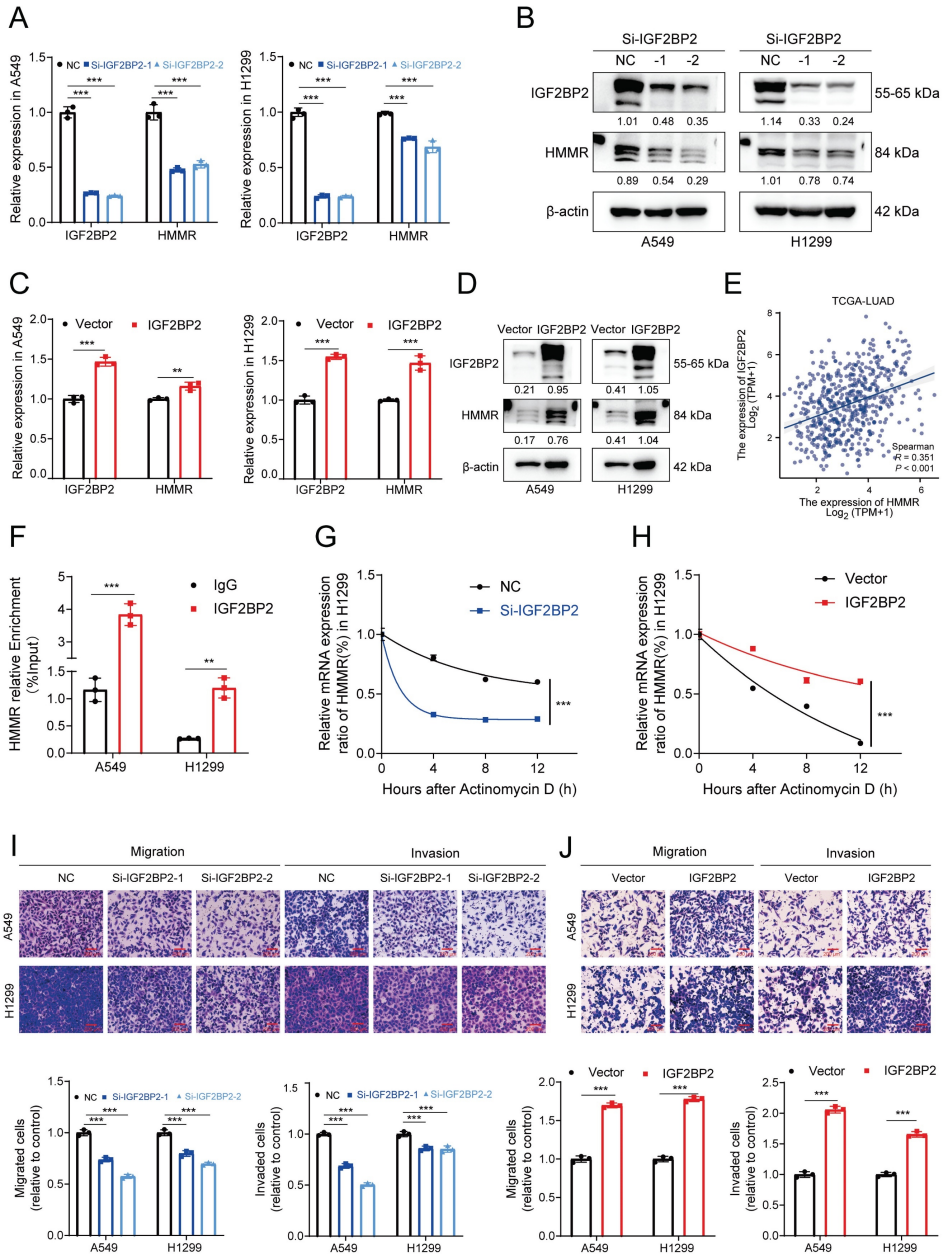
** IGF2BP2 enhances HMMR expression by stabilizing HMMR mRNA.** (A and B) Relative mRNA and protein levels of HMMR in NSCLC cell lines with IGF2BP2 knockdown. (C and D) Relative mRNA and protein levels of HMMR in NSCLC cell lines overexpressing IGF2BP2. (E) Correlation analysis between HMMR and IGF2BP2 expression levels in LUAD. (F) RNA immunoprecipitation (RIP) assay with anti-IGF2BP2 antibody or IgG to detect binding to HMMR in A549 and H1299 cells; IgG served as a negative control. (G and H) Relative mRNA expression levels of HMMR analyzed by qRT-PCR in actinomycin D-treated H1299 cells at 0, 4, 8, and 12 hours. (I and J) Transwell assay images illustrating the mobility and invasiveness of NSCLC cells following IGF2BP2 overexpression or knockdown. All data are presented as mean ± SD from three independent experiments.

To explore m^6^A modifications in NSCLC, a human m^6^A epitranscriptomic microarray was conducted, and the findings revealed that compared to normal tissues, lung cancer ones harbored hypermethylated and modified HMMR (Figure S3A). This increase in HMMR expression in tumor tissues suggests that an m^6^A-binding protein may positively regulate HMMR through methylation. YTHDF1 is known to facilitate mRNA translation, while the IGF2BP family, including IGF2BP2, is involved in mRNA stabilization. Following the removal of the most prevalent “readers,” qRT-PCR analysis revealed that HMMR mRNA expression was significantly decreased after knocking down IGF2BP2 (Figure S3B). To investigate IGF2BP2's functions in cell migration and invasion, we created IGF2BP2-knockdown and IGF2BP2-overexpressing cell lines. Results indicated that migration and invasion were reduced in IGF2BP2-knockdown cells (Figure 6I, S3D) and enhanced in IGF2BP2-overexpressing cells (Figure 6J, S3C). Consistent with this, IGF2BP2 knockdown resulted significantly reduced both the mRNA and protein levels of HMMR (Figure 6A-B), while IGF2BP2 overexpression increased these levels (Figure 6C-D). The TCGA-LUAD database further showed that HMMR and IGF2BP2 expressions were positively correlated (Figure 6E). RNA stability assays showed that IGF2BP2 overexpression stabilizes HMMR mRNA, whereas its knockdown decreases mRNA stability (Figure 6G-H). RIP assays using anti-IGF2BP2 antibodies confirmed that IGF2BP2 specifically binds to HMMR in both A549 and H1299 cells (Figure 6F), highlighting IGF2BP2's crucial role in HMMR's m^6^A modification. In conclusion, the results demonstrate that, in NSCLC, IGF2BP2 is crucial for promoting NSCLC cell migration and invasion by stabilising HMMR mRNA and upregulating its expression.

### ALKBH5-mediated upregulation of HMMR via IGF2BP2-dependent m^6^A modification

To further investigate this phenomenon, we conducted MeRIP and analyzed the resulting products using qRT‒PCR. Utilizing SRAMP, we identified six potential m^6^A modification sites in HMMR mRNA and designed specific m^6^A primers for MeRIP qRT‒PCR (Figure S3E).

**Figure 7:**
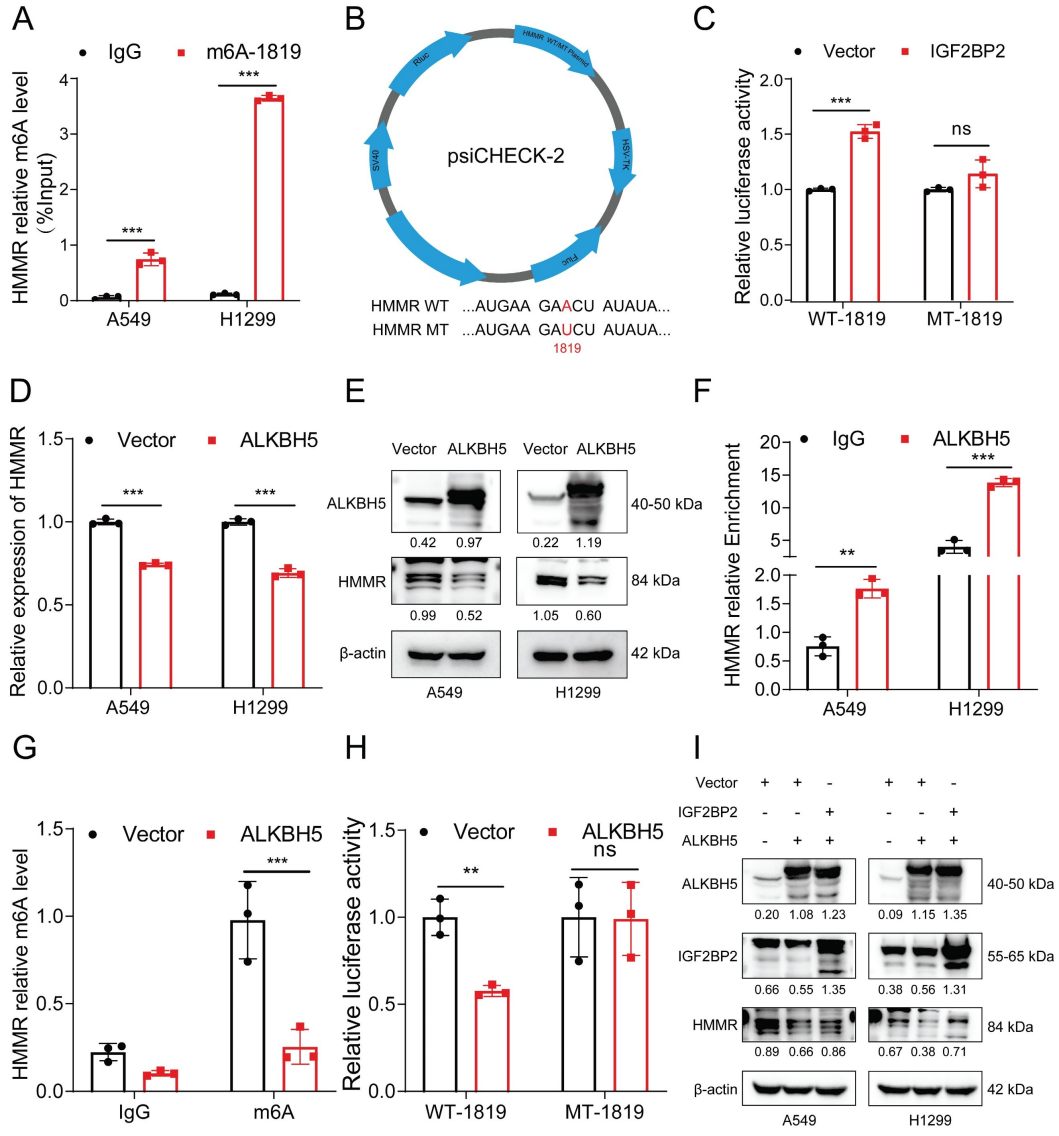
** ALKBH5-mediated upregulation of HMMR via IGF2BP2-dependent m^6^A modification.** (A) MeRIP qRT-PCR assay using an anti-m^6^A antibody or IgG to assess binding to HMMR in A549 and H1299 cells; IgG served as a negative control. (B) Schematic representation of HMMR-WT (wild type) and HMMR-MT (mutant type) sequences. (C) Relative luciferase activity of wild-type and mutant HMMR reporter vectors in IGF2BP2-overexpressing NSCLC cells. (D and E) Relative mRNA and protein levels of HMMR in ALKBH5-overexpressing NSCLC cell lines. (F) RIP assay with anti-ALKBH5 antibody or IgG to detect binding to HMMR in A549 and H1299 cells; IgG was used as a negative control. (G) The m^6^A level of m^6^A in HMMR in A549 cells with ALKBH5 overexpression was analysed by MeRIP-qPCR. (H) Luciferase reporter assays measuring the luciferase activities of HMMR WT and HMMR Mut in H1299 cells overexpressing ALKBH5. (I) Overexpression of IGF2BP2 rescued the ALKBH5-induced downregulation of HMMR in NSCLC cells.

The MeRIP qRT‒PCR assay revealed significant enrichment of HMMR mRNA by the anti-m^6^A antibody in both H1299 and A549 cells (Figure 7A). Among these sites, #1819 emerged as the most reliably modified region in the HMMR transcript (Figure 7A). To further elucidate the role of this modification, we replaced the N^6^-methylated adenosine (A) at site #1819 with uracil (U) in the m^6^A consensus sequence of HMMR mRNA, generating a HMMR mutant resistant to m^6^A modification (Figure 7B). As shown by the luciferase reporter assays, cells overexpressing wild-type HMMR exhibited a significant increase in luciferase activity in comparison with mutant ones, where this increase was nearly abolished (Figure 7C). This suggests that HMMR expression modulation is regulated by m^6^A modification at site #1819. Considering the hypermethylation observed in lung cancer tissues relative to paraneoplastic tissues, we hypothesized that demethylases might be expressed at lower levels in lung cancer tissues. To test this, we analyzed the expression of the demethylation enzymes ALKBH5 and FTO in lung adenocarcinoma and normal tissues using the TCGA database. Our results revealed low expression levels of both ALKBH5 and FTO in lung cancer tissues (Figure S3F), with ALKBH5 being the sole enzyme showing negative regulation of HMMR (Figure S3G-H). Consistent with this, overexpression of ALKBH5 significantly reduced the mRNA and protein levels of HMMR (Figure 7D-E). Additionally, RIP assays confirmed that ALKBH5 specifically binds to HMMR in both A549 and H1299 cells (Figure 7F). We further performed MeRIP in ALKBH5-overexpressing cells. The m^6^A modification of HMMR mRNA was significantly reduced after ALKBH5 overexpressed (Figure 7G, S3I). Luciferase reporter assays further showed that, in ALKBH5-overexpressing cells harboring wild-type HMMR, the luciferase activity was significantly lower, while this decrease was nearly abolished in the mutant cells (Figure 7H). Rescue experiments aimed at establishing the regulatory axis of ALKBH5/IGF2BP2/HMMR in NSCLC demonstrated that transfection of the ALKBH5 plasmid into IGF2BP2-overexpressing cells restored HMMR levels (Figure 7I). In summary, these findings indicate that ALKBH5 mediates the upregulation of HMMR through an IGF2BP2-dependent m^6^A modification mechanism.

## Discussion

**Figure 8:**
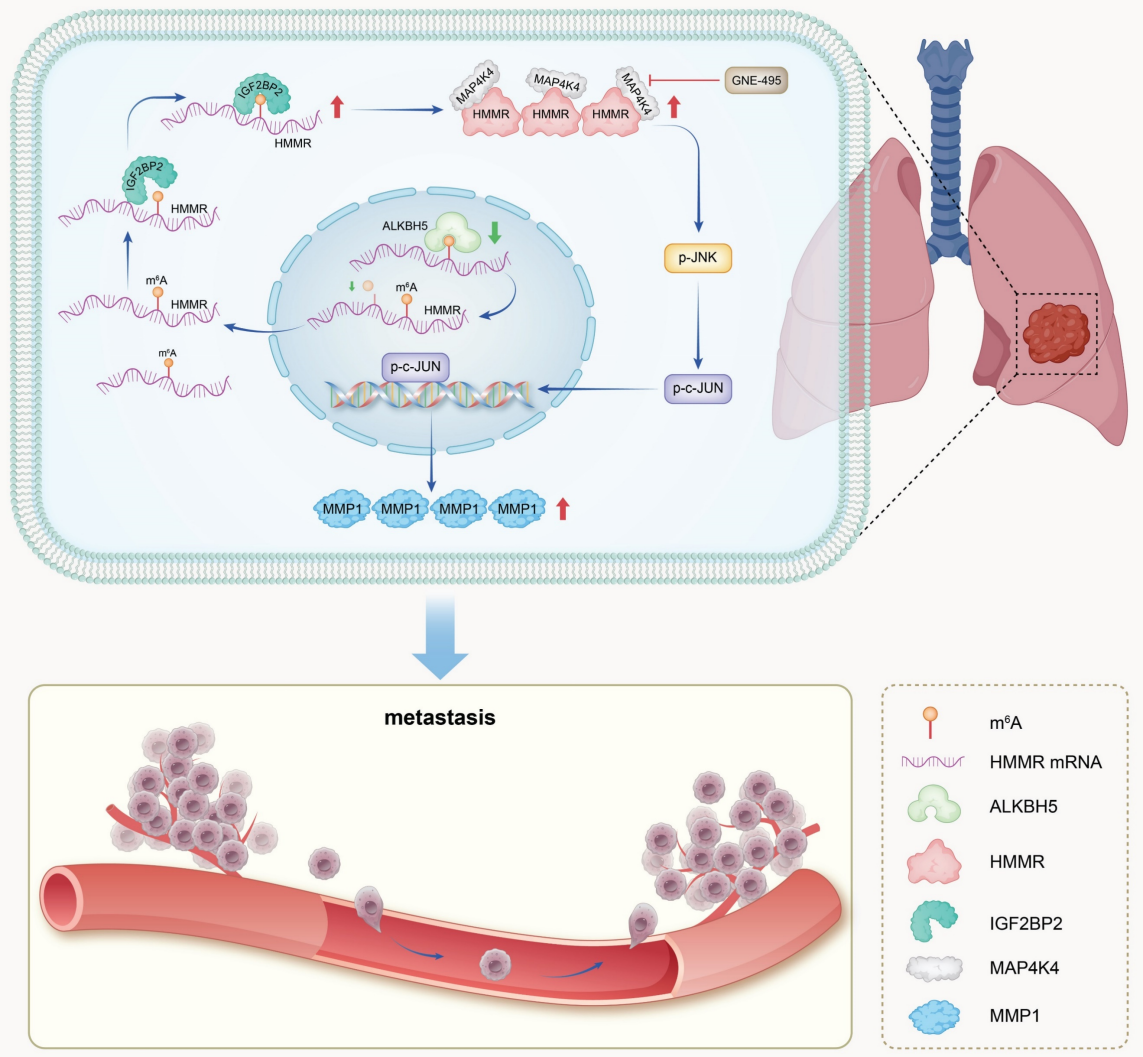
** Schematic model.** In summary, IGF2BP2 directly binds to the m^6^A site within the CDS region of HMMR mRNA, enhancing mRNA stability and leading to increased HMMR expression. Elevated HMMR then interact with MAP4K4, which activates the p-JNK/p-c-JUN/MMP1 signaling cascade, thereby promoting metastasis in NSCLC cells.

In NSCLC patients, metastasis remains the primary driver of poor prognosis(26. Despite advancements in various treatments^27^, patients with advanced NSCLC still have a 5-year overall survival rate of less than 30%^28^,^29^. This is largely due to the complex and not yet fully understood pathogenic mechanisms and metastatic processes of NSCLC^8^. Therefore, exploring the molecular mechanisms associated with metastatic disease and identifying novel, effective therapeutic targets is crucial. By studying the functions of HMMR, the current study identified it as a possible prognostic biomarker for NSCLC and established a link between it and metastasis. Our findings indicate that overexpression of HMMR in NSCLC cells is essential for multiple stages of the metastatic process, including the upregulation of MMP1 as well as enhanced cell motility and invasiveness. Mechanistically, HMMR promotes tumor metastasis by interacting with MAP4K4, which in turn activates the p-JNK/p-c-JUN/MMP1 signaling pathway. Notably, our mouse model experiments demonstrated that the MAP4K4 inhibitor GNE-495 effectively hindered lung metastasis induced by HMMR overexpression. Additionally, our data reveal that, by binding to HMMR's m^6^A site, IGF2BP2 enhances the stability of its mRNA and increases its expression levels. These findings provide novel insights into the molecular mechanisms driving cancer metastasis (Figure 8).

HMMR serves as a receptor for hyaluronic acid^30^ and is engaged in various biological processes, including maintenance of stem cell characteristics^9^, cellular proliferation^31^, and metastasis^32^,^33^. Research has shown that HMMR is frequently overexpressed in numerous cancers and is linked to adverse clinical features and poor prognosis^18^,^34^. In consistence with the findings reported in the existing literature, our study demonstrates a significant overexpression of HMMR in NSCLC tissues and a strong correlation between elevated HMMR levels and unfavorable pathological features, such as advanced N classification, M stage, decreased OS probability and poor PFS. HMMR also has emerged as a highly independent prognostic factor, with its efficacy demonstrated through the nomogram for predicting one-, three-, and five-year survival probabilities. The results suggest that HMMR could represent a potential prognostic biomarker of NSCLC and may influence its metastatic potential.

To elucidate the mechanism by which HMMR facilitates tumor metastasis, we conducted mRNA sequencing and mass spectrometry, identifying MAP4K4 as a key protein that interacts with and is regulated by HMMR. MAP4K4, a serine/threonine protein kinase, plays a critical role in embryonic development^35^, systemic inflammation^36^, and various pathological conditions, including diabetes^37^ and atherosclerosis^38^. Additionally, MAP4K4 has been implicated in tumor progression and is considered a negative prognostic factor in several cancers, including prostate cancer^39^, lung adenocarcinoma^40^, and hepatocellular cancer^41^. Huang *et al.* found that the transcription factor SOX6 regulates MAP4K4 expression, which mediates SOX6-induced autophagy and reduces chemotherapeutic sensitivity in cervical cancer^42^. Chen *et al.* demonstrated that MAP4K4 contributes to the phosphorylation of ADAM10 at Ser436, preventing N-cadherin degradation and promoting abdominal metastasis in ovarian cancer. Moreover, administration of the MAP4K4 inhibitor GNE-495 effectively suppressed abdominal metastasis in tumors^43^. Guo *et al.* showed that HMMR interacts with AURKA, inhibiting the ubiquitination degradation of AURKA and thereby activating the mTORC2/AKT signaling pathway, which in turn promotes metastasis of prostate cancer^19^. The aforementioned literature provides compelling evidence that both HMMR and MAP4K4 play pivotal roles in promoting distant tumor metastasis. Our research has uncovered a novel link between HMMR and MAP4K4. We found that HMMR knockdown reduces MAP4K4 expression, whereas HMMR overexpression increases MAP4K4 levels. This modulation significantly affects downstream signaling: HMMR knockdown inhibits the phosphorylation of JNK (p-JNK) and c-JUN (p-c-JUN), while HMMR overexpression activates these signaling pathways. We also observed that HMMR interacts with MAP4K4, confirming its key role in regulating the JNK signaling pathway. Furthermore, the activation of the p-JNK/p-c-JUN pathway was effectively suppressed by MAP4K4 inhibitors, such as GNE-495 or siRNA targeting MAP4K4, thereby inhibiting the cellular behaviors associated with migration and invasion driven by HMMR overexpression. Additionally, c-JUN was found to regulate the expression of MMP1, a known mediator of cancer invasiveness, and MAP4K4, creating a feedback loop that may enhance metastatic potential. The identification of the HMMR/MAP4K4/p-c-JUN/MMP1 signaling axis provides new insights into NSCLC metastasis and could inform future therapeutic strategies aimed at inhibiting NSCLC progression.

The regulation of gene expression encompasses a range of processes, including epigenetic regulation, transcriptional regulation, post-transcriptional modification, translational regulation, and post-translational modification. In a study by Rozengurt *et al.*, it was demonstrated that YAP/TAZ promotes HMMR transcription and correlates with poor prognosis in pancreatic cancer^44^. Similarly, Wang *et al.* showed that YAP/TEAD promotes HMMR transcription and metastasis in breast cancer^45^. He and colleagues observed that CHOP promotes the transcriptional expression of HMMR and undergoes degradation by TRIM29-mediated ubiquitination in the progression of hepatitis B virus (HBV)-induced hepatitis to hepatocellular carcinoma^13^. In the present study, we have identified a post-transcriptional modification that regulate the increased expression of HMMR in patients with lung cancer. Human m^6^A epitranscriptomic microarray data indicated that, compared to normal tissues, lung cancer ones had significantly elevated m^6^A modification levels in their HMMR mRNA. IGF2BP2, identified as a novel m^6^A reader, has been shown to enhance the stability and translation of mRNA. It is involved in a number of biological processes, such as stemness maintenance^46^, cell proliferation^47^ and metastasis^48^. Lin *et al.* have shown that IGF2BP2 upregulate EREG expression and promote the progression of oral cancer via the FAK/Src signaling pathway^49^. Liu *et al.* demonstrated that IGF2BP2/3 bind to EphA2 and VEGFA, which have been modified by METTL3, to prevent their mRNA degradation and promote vasculogenic mimicry (VM) formation, thereby facilitating the progression of colorectal cancer^50^. Fang *et al.* provided evidence that IGF2BP2 is mainly secreted by exosomes in LUAD and taken up by endothelial cells, which increases the RNA stability of FLT4 to promote angiogenesis and metastasis^51^. Our study reveals that IGF2BP2 knockdown leads to decreased expression of HMMR, while its overexpression results in HMMR upregulation. To determine how IGF2BP2 regulates HMMR, RIP-qPCR was performed, with the results identifying HMMR as a direct mRNA target of IGF2BP2. The m^6^A readers are known to influence mRNA stability, and our Actinomycin D experiment supports the hypothesis that IGF2BP2 knockdown reduces HMMR mRNA stability, whereas IGF2BP2 overexpression increases it. To confirm that IGF2BP2 enhances HMMR expression through m^6^A modification, we performed Me-RIP experiments, which detected N^6^-methyladenosine modification in HMMR mRNA. Subsequent analysis with SRAMP software, a reliable sequence-based m^6^A site predictor, identified high-confidence m^6^A sites within the CDS region of HMMR mRNA. Specifically, m^6^A modification was observed at site 1819 in both A549 and H1299 cell lines. Luciferase reporter assays validated that IGF2BP2 regulates HMMR mRNA expression through m^6^A modification. In summary, our findings indicate that IGF2BP2 acts directly on HMMR mRNA by binding to the m^6^A site of its CDS region, thereby enhancing the stability of the mRNA and increasing HMMR expression.

This study also has some limitations. First, the precise mechanism underlying the interaction between HMMR and MAP4K4 requires further investigation. Second, while it is clear that the total proteins JNK and c-JUN are altered in response to JNK signaling pathway activation or inhibition, additional research is required to gain a comprehensive understanding of the extent of these changes.

## Conclusions

This study found that, in NSCLC, cell migration and invasion was enhanced by HMMR which acts as an oncogene. Furthermore, the stability and expression of HMMR mRNA was increased when IGF2BP2 binds directly to the m^6^A site of its CDS region. The results also provide the first evidence that HMMR facilitates metastasis through the JNK signaling pathway by interacting with MAP4K4, suggesting that this interaction could be a promising target for NSCLC treatment.

## Supplementary Material

Supplementary figures and tables.

## Figures and Tables

**Table 1:** Association between HMMR expression and clinicopathological characteristics of patients with LUAD

Characteristics	Low expression of HMMR	High expression of HMMR	*P* value
Pathologic T stage, n (%)			0.436
T1&T2	237 (44.2%)	231 (43.1%)	
T3&T4	31 (5.8%)	37 (6.9%)	
Pathologic N stage, n (%)			< 0.001^***^
N0	195 (37.3%)	155 (29.6%)	
N1&N2&N3	62 (11.9%)	111 (21.2%)	
Pathologic M stage, n (%)			0.007^**^
M0	175 (44.9%)	190 (48.7%)	
M1	5 (1.3%)	20 (5.1%)	
Pathologic stage, n (%)			0.002^**^
Stage I&Stage II	224 (42.2%)	197 (37.1%)	
Stage III&Stage IV	40 (7.5%)	70 (13.2%)	
Gender, n (%)			0.006^**^
Female	160 (29.7%)	129 (23.9%)	
Male	109 (20.2%)	141 (26.2%)	
Age, n (%)			0.220
<= 65	122 (23.5%)	135 (26%)	
> 65	139 (26.7%)	124 (23.8%)	
OS event, n (%)			< 0.001^***^
Alive	196 (36.4%)	151 (28%)	
Dead	73 (13.5%)	119 (22.1%)	
PFS event, n (%)			0.040^*^
No	168 (31.2%)	145 (26.9%)	
Yes	101 (18.7%)	125 (23.2%)	

The *P* value was measured by Chi-square test. *P<0.05, **P<0.01, ***P<0.001.

## Abbreviations

NSCLC: Non-small cell lung cancer
HMMR: Hyaluronan Mediated Motility Receptor
MAP4K4: Mitogen-Activated Protein Kinase Kinase Kinase Kinase 4
IGF2BP2: Insulin Like Growth Factor 2 mRNA Binding Protein 2
ALKBH5: alkB homolog 5, RNA demethylase
FTO: Fat mass and obesity‑associated protein
MMP1: matrix metallopeptidase 1
