# Supplementary material for: N6-methyladenosine Reader IGF2BP2-modified HMMR Promotes Non-small Cell Lung Cancer Metastasis via Interaction with MAP4K4
Source: Int J Biol Sci. 2025 Jan 21;21(4):1391–409. doi: 10.7150/ijbs.104097 (PMC11844294; doi:10.7150/ijbs.104097)
Supplement: Supplementary file 1 — Supplementary figures and tables. [file ijbsv21p1391s1.pdf]

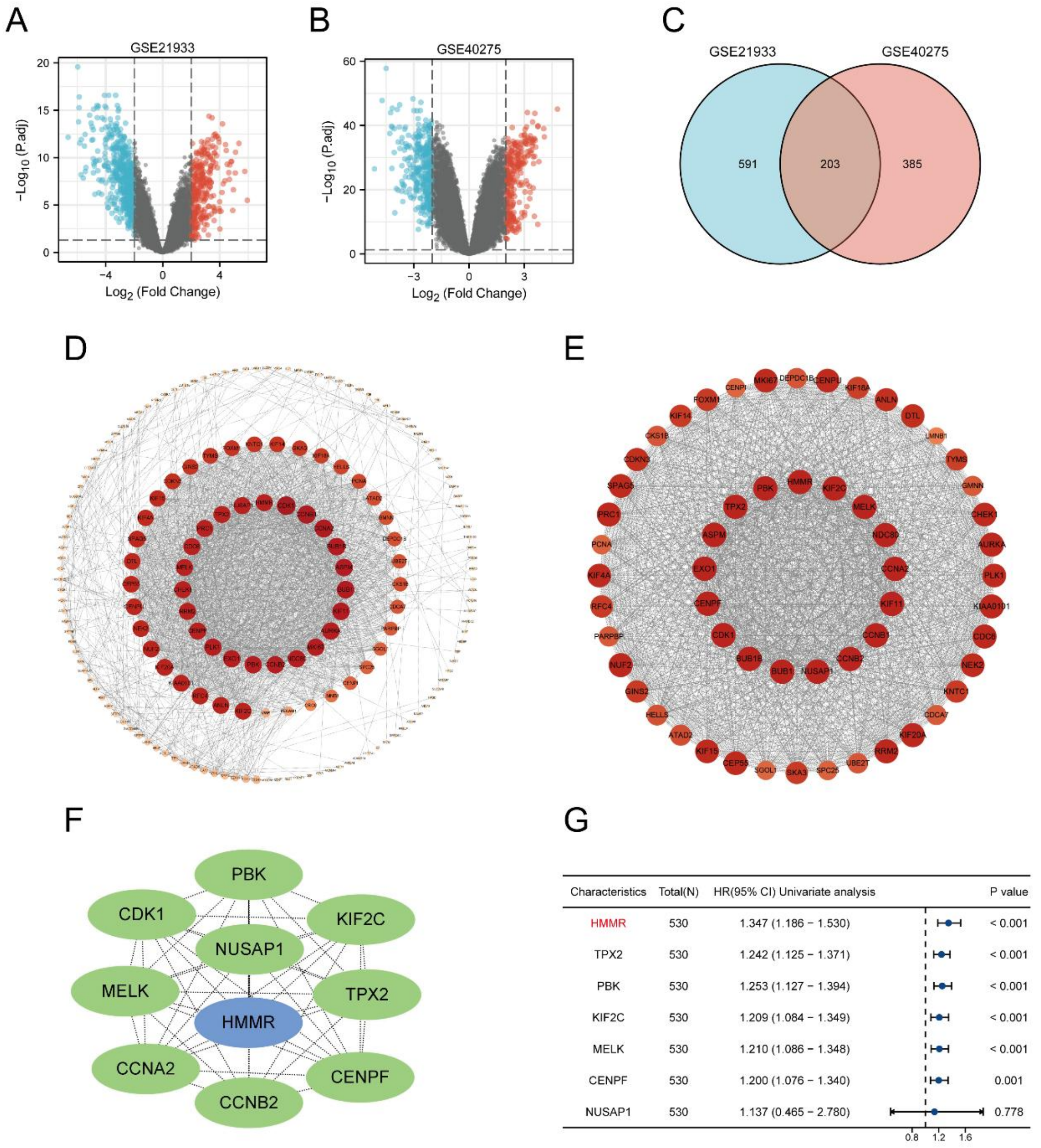

Figure S1 Identification of HMMR as a Candidate Target Gene. (A) Volcano plot of GSE21933. (B) Volcano plot of GSE40275. (C) Venn diagrams of overlapped DEGs. (D) PPI network of overlapped DEGs. (E) Genes in the module with the highest MCODE value. (F) Top 10 hub genes. (G) Univariate Cox regression analysis of HMMR to identify prognostic factors.

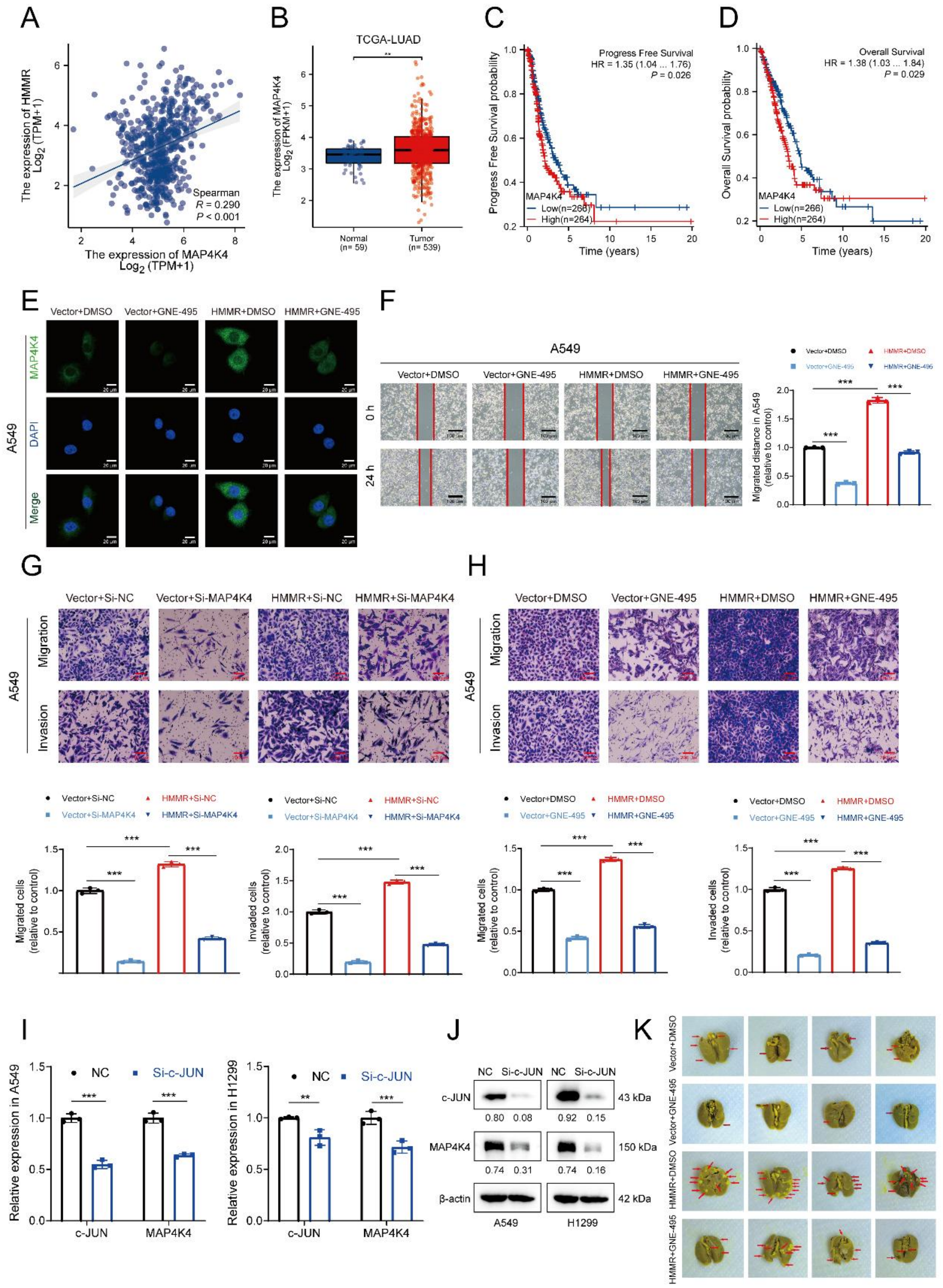

Figure S2 Expression and Clinical Significance of MAP4K4 in LUAD. (A) Correlation analysis between HMMR and MAP4K4 expression levels in LUAD. (B) The TCGA database was utilized to compare the mRNA expression levels of

MAP4K4 in LUAD tissues relative to normal lung tissues. (C and D) Analysis of progression-free survival (PFS) and overall survival (OS) probabilities in LUAD patients with high versus low MAP4K4 expression, using the median expression level of MAP4K4 as the cut-off value. (E) Immunofluorescence staining of MAP4K4 in HMMR-overexpressing and control cells, with or without MAP4K4 inhibitor GNE-495. (F) The effect of MAP4K4 inhibitor GNE-495 on HMMR-induced migration enhancement. (G and H) The impact of MAP4K4 knockdown or GNE-495 treatment on HMMR-induced migration and invasion. (I) qRT-PCR analysis of MAP4K4 expression following si-c-JUN transfection. (J) Western blot analysis of MAP4K4 expression after si-c-JUN transfection. (K) Photographs showing lung metastatic nodules in mice following injection of A549 cells overexpressing HMMR or the control vector, with treatment by DMSO or GNE-495. Metastatic nodules are indicated by red arrowheads.

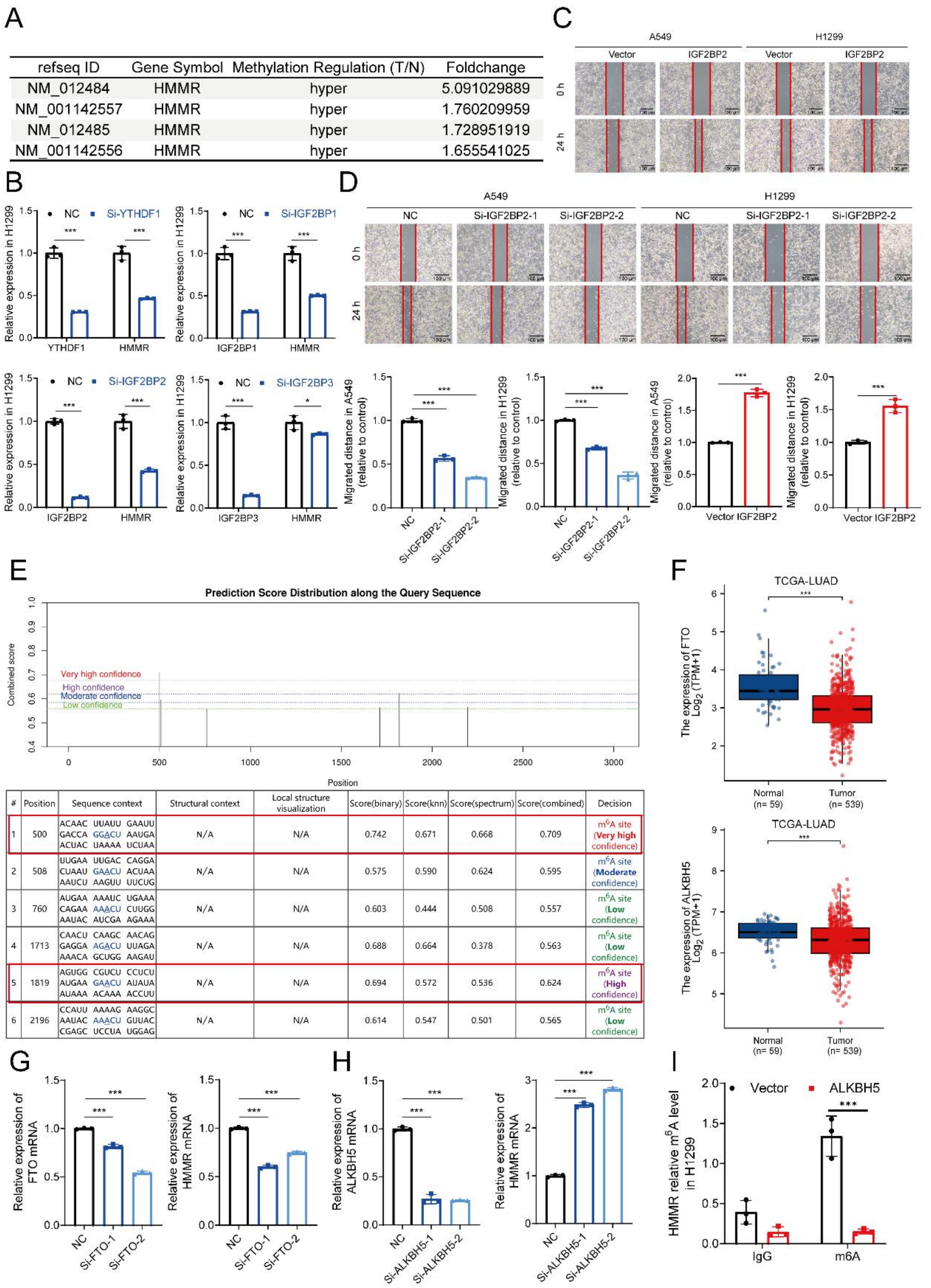

Figure S3 Screening of Demethylases and Readers for HMMR m^{6}A Modification.

(A) Comparative analysis of hypermethylation and modifications in HMMR within lung cancer tissue versus normal tissue using a human m^{6}A epitranscriptomic

microarray. (B) qRT-PCR analysis of HMMR expression following transfection with si-YTHDF1, si-IGF2BP1, si-IGF2BP2, si-IGF2BP3. (C and D) Wound healing images depicting the mobility of NSCLC cells after overexpression or knockdown of IGF2BP2. (E) SRAMP prediction highlighting putative m^6A modification sites within HMMR mRNA. (F) Evaluation of FTO and ALKBH5 mRNA expression in unpaired LUAD tissue versus normal lung tissue within the TCGA database. (G and H) qRT-PCR analysis of HMMR expression after transfection with si-FTO or si-ALKBH5. (I) The m^6A level of m^6A in HMMR in H1299 cells with ALKBH5 overexpression was analysed by MeRIP-qPCR.

Table S1. The sequences of genes used in assays of siRNA knockdown.

Target Gene Name	siRNA sequences (5' to 3')
NC (negative control)	UUCUCCGAACGUGUCACGUTT
Si-HMMR-1	UCUCCUCUAUGAAGAACUATT
Si-HMMR-2	CAACUCAAAUCGGAAGUAUTT
Si-MAP4K4	CUUCCUGUUCCUCUUAUAUTT
Si-c-JUN	GACCUUAUGGCUACAGUAATT
Si-FTO-1	ACCAAGGAGACUGCUAUUUTT
Si-FTO-2	GUGGCAGUGUACAGUUAUATT
Si-ALKBH5-1	AAGGCUGUUGGCAUCAAUATT
Si-ALKBH5-2	ACCCAGCUAUGCUUCAGAUTT
Si-YTHDF1-1	GGACAGUCAAAUCAGAGUATT
Si-IGF2BP1-1	AAAUAAUGAAGAAAGUUCGTT
Si-IGF2BP2-1	GAGAUAGAGAUUAUGAAGATT
Si-IGF2BP2-2	UGAAGCUGGAAGCGCAUAUTT
Si-IGF2BP3-1	GGUGAAUGAACUUCAGAAUTT

Table S2. Primer sets used for qRT-PCR analysis.

Gene	Forward Primer (5' to 3')	Reverse Primer (5' to 3')
HMMR	ACCAACTCAAGCAACAGGAGGAAG	TTCTTCATAGAGGAGACGCCACTTG
MAP4K4	CAACATCTCGCTCCCCTGTT	CCTGGGCTCAATACTGGTGG
c-JUN	AGAACTCGGACCTCCTCACCTC	ATGTGCCCGTTGCTGGACTG
MMP1	TTACACGCCAGATTTGCCAAGAG	TCAGAGGTGTGACATTACTCCAGAG
YTHDF1	ACCTGTCCAGCTATTACCCG	TGGTGAGGTATGGAATCGGAG
IGF2BP1	GATGAAGGCCATCGAAACTTTC	GGGGTGGAATATTTCGGATTTG
IGF2BP2	GACAGGTCCTGCTGAAGTCC	CGCAGCGGGAAATCAATCTG
IGF2BP3	GAGGCGCTTTCAGGTAAAATAG	AATGAGGCGGGATATTTCGTAT
FTO	GGATGCTGTGCCATTGTGTATGTC	TCCACTTCATCTTGTCCGTTGTAGG
ALKBH5	TGCAAGCTCATGCAAACACC	GACCCAACGTGGCAAGTCTA
β-actin	CCTGGCACCCAGCACAAT	GGGCCGGACTCGTCATAC

Table S3. Primer sets used for Me-RIP qRT-PCR analysis.

Gene	Forward Primer (5' to 3')	Reverse Primer (5' to 3')
HMMR(#500)	TTGACCAGGACTAATGAACTACTAA	TCGAGACTCCTTTGGGTGAC
HMMR(#1819)	GGCGTCTCCTCTATGAAGAACTA	AGCTGACAGCGGAGTTTTGA
